# Beneficial effect of statin on preventing contrast-induced acute kidney injury in patients with renal insufficiency: A meta-analysis

**DOI:** 10.1097/MD.0000000000019473

**Published:** 2020-03-06

**Authors:** AJin Cho, Young-Ki Lee, Seo Young Sohn

**Affiliations:** a Division of Nephrology, Department of Internal Medicine, Hallym University College of Medicine and Hallym University Kidney Research Institute, Hallym University Medical Center, Seoul, Korea; b Division of Endocrinology and Metabolism, Department of Internal Medicine, Myongji Hospital, Hanyang University College of Medicine, Goyang, Korea.

**Keywords:** acute kidney injury, contrast media, statin

## Abstract

**Background:**

Renal insufficiency is an important predictor of contrast-induced acute kidney injury (CI-AKI). We performed a meta-analysis to examine the effects of short-term statin therapy on the incidence of CI-AKI, particularly in patients with renal insufficiency.

**Methods:**

A systematic search was conducted to retrieve randomized controlled trials (RCTs) that investigated the impact of statin pretreatment before administration of contrast media on the development of CI-AKI in patients with mild to moderate renal insufficiency. The primary outcome was development of CI-AKI. The secondary outcome was the incidence ofacute kidney injury requiring hemodialysis.

**Results:**

Data analysis from 8 RCTs, which included a total of 2313 subjects in the statin-treated group and 2322 in the control group, showed that statin pretreatment was associated with significant reduction of the risk of CI-AKI (relative risk [RR] = 0.59; 95% confidential interval [CI] 0.44–0.79; *P* = .0003, *I*^2^ = 0%). A beneficial effect of statin on preventing CI-AKI was consistent, regardless of the dose of statin and use of N-acetylcysteine. In subgroup analysis based on baseline estimated glomerular filtration rate (eGFR), patients with baseline eGFR <60 mL/min/1.73 m^2^ (RR = 0.63; 95% CI 0.41–0.98; *P* = .04, *I*^2^ = 0%) and 30 < eGFR < 90 mL/min/1.73 m^2^ (RR = 0.56; 95% CI 0.39–0.82; *P* = .003, *I*^2^ = 0%) showed significant reduction of risk of CI-AKI.

**Conclusion:**

Statin pretreatment is effective at preventing CI-AKI and should be considered in patients with preexisting renal insufficiency.

## Abstract 2

Supplemental Digital Content is available in the text

## 1 Introduction

Contrast-induced acute kidney injury (CI-AKI) is common and an important complication caused by contrast media administration during diagnostic or interventional cardiovascular procedures. CI-AKI is the third leading cause of hospital-acquired acute kidney injury, accounting for 12% of all cases.^[1]^ Although the incidence of CI-AKI is relatively low in patients with normal renal function, it is dramatically higher in patients with preexisting renal insufficiency.^[2,3]^ Other risk factors for CI-AKI include older age, diabetes mellitus (DM), use of large contrast media doses, the concurrent use of nephrotoxic drugs, hemodynamic instability, and other comorbidities.^[4]^

Because CI-AKI is associated with prolonged hospitalization, increased morbidity and deaths, and increased healthcare cost,^[4]^ various strategies have been used to prevent CI-AKI in at-risk populations.^[5]^ Among those approaches, increasing evidence has suggested that statins play a role in preventing CI-AKI by its pleiotropic effects, which include anti-inflammatory, antioxidant, and antithrombotic actions.^[6,7]^ All these effects counteract pathophysiologic mechanisms that promote the development of CI-AKI.

Previous meta-analyses focused on the effect of statin for the prevention of CI-AKI have been published,^[8–11]^ but they have not shown whether statin is effective in patients with preexisting renal insufficiency, who are prone to a higher risk of CI-AKI development. Therefore, the purpose of this meta-analysis was to examine the effects of short-term statin therapy on the incidence of CI-AKI, particularly in patients with preexisting chronic kidney disease (CKD) undergoing coronary angiography or percutaneous coronary intervention (PCI).

## 2 Methods

### 2.1 Data sources and search strategy

This meta-analysis was performed in accordance with the Preferred Reporting Items for Systemic Reviews and Meta-Analyses (PRISMA) recommendations. We conducted electronic searches in the MEDLINE, EMBASE, Cochrane library, and Web of Science on April 20, 2019. In our literature search, there were no restrictions on language, study period, or sample size. The following keywords were used: “Hydroxymethylglutaryl-CoA Reductase Inhibitors," “Simvastatin," “Atorvastatin Calcium," “Rosuvastatin Calcium," “Coronary Angiography," “Contrast Media," “Angiography," “Acute Coronary Syndrome," and “Percutaneous Coronary Intervention." Electronic database searches used both free-text words and Medical Subject Headings. We also searched gray literature from the website of Open Gray. The specific searching strategy is described in the supplemental file (Supplementary file). Our institution approved this study (IRB No: 2019-04-005).

### 2.2 Study selection

Two independent reviewers (A.C. and S.Y.S.) screened the titles and abstracts of all retrieved records for eligibility. Studies that were clearly irrelevant were excluded at this stage. Any disagreements were resolved by a third author (Y.K.L.). Studies were included if they met the following criteria: randomized controlled trials (RCTs) investigating the efficacy of statin in preventing CI-AKI before coronary angiography or PCI. The intervention was statin vs placebo or no statin treatment. In cases where concomitant prophylactic strategies were used (such as N-acetylcysteine [NAC], sodium bicarbonate, or other preventive medications), both arms need to have shared the same concomitant prophylactic measures. No statin type and dosage limitations were imposed; RCTs performed only in patients with mild to moderate renal insufficiency, CKD stage 2 to 4; CI-AKI reported or defined as an increase in serum creatinine (SCr) ≥0.5 mg/dL (44.2 mmol/L) or a 25% elevation of SCr above baseline within 5 days after contrast media exposure. We excluded the followings: animal studies; nonrandomized studies; studies that included subjects with estimated glomerular filtration rate (eGFR) >90 mL/min and end-stage renal disease requiring dialysis; studies comparing the high-dose statin vs low-dose statin without other control groups; and duplicated datasets.

### 2.3 Study outcomes

The primary outcome was the development of CI-AKI, which was defined as an absolute increase of SCr of 0.5 mg/dL or an increase in baseline SCr level of 25% within 24 hours to 5 days after exposure to the contrast medium. The secondary outcome was the need for hemodialysis within 5 days after administration of a contrast medium.

### 2.4 Data extraction and quality assessment

We independently carried out data extraction on the characteristics of the studies (study design, sample size, type of contrast media, inclusion criterion), clinical characteristics of the patients (age, sex, and DM and baseline SCr), procedural characteristics, hydration protocol, and incidence of CI-AKI. The quality of the included studies was evaluated by using the Revised Cochrane risk of bias tool for randomized trials (RoB 2.0). This assessment was made independently by 2 reviewers (A.C. and S.Y.S.), and disagreements were resolved by a third reviewer (Y.K.L.) through consensus.

### 2.5 Statistical analyses

The primary endpoint, development of CI-AKI, was quantified and reported as pooled relative-risk (RR) ratio with a 95% confidence interval. The meta-analysis was conducted by combining the RR of individual studies into a pooled RR using a random-effects model; *P* < .05 was considered statistically significant. We tested for heterogeneity using the Chi-squared test, *I*^2^ test, and Tau^2^ test. Funnel plots were performed to subjectively assess for publication bias. All statistical analyses were performed by using Review Manager Version 5.3, which was supplied by the Cochrane Collaboration.

## 3 Results

### 3.1 Search results

**Figure 1:**
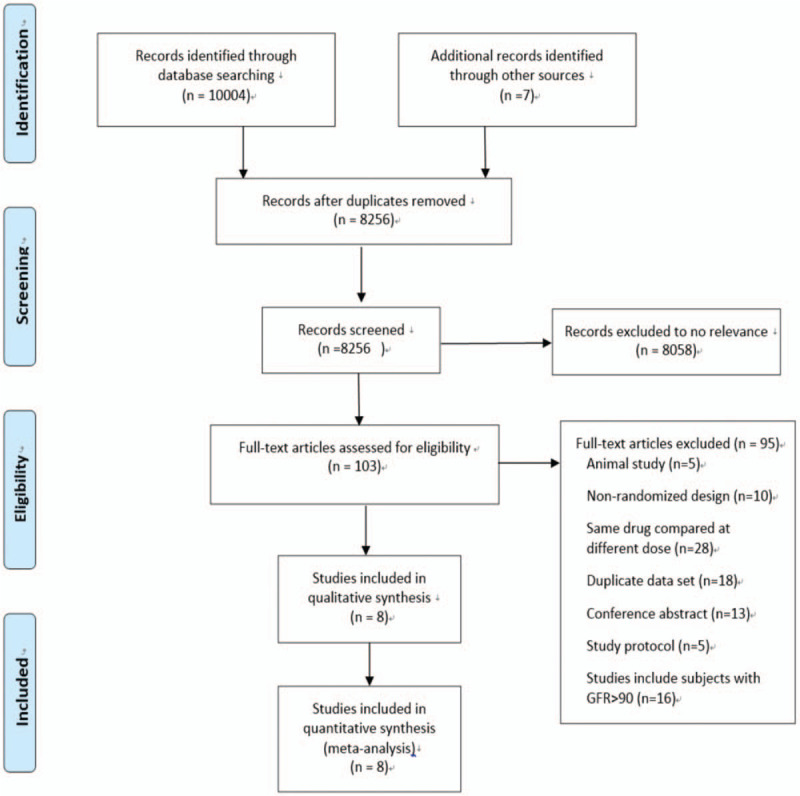
Flow chart of the selection for eligible studies.

Figure 1 shows our search strategy and identified 10,011 potentially relevant citations. We also searched Gray literature from the website of Open Gray, but could not find any abstract that met our inclusion criteria. After excluding duplications, 8256 records remained. Titles and abstracts of these records were screened for inclusion. Then 8154 articles were excluded because of irrelevance of topics and study types. The full texts of 103 articles were reviewed, and 8 RCTs met the inclusion criteria.^[12–19]^

### 3.2 Study characteristics and intervention

Baseline procedural characteristics are shown in Table 1. Most of the included studies^[13–19]^ were on patients undergoing planned coronary angiography or PCI. Two studies^[12,18]^ included patients undergoing peripheral angiography. Four studies^[12,13,16,17]^ included patients with an estimated creatinine clearance <60 mL/min or an eGFR <60 mL/min/1.73 m^2^, and 3 studies^[14,18,19]^ included patients with 30 ≤ eGFR < 90 mL/min/1.73 m^2^. In 4 studies,^[13–16]^ patients were treated with atorvastatin 80 mg, in 2 studies^[18,19]^ with rosuvastatin 10 mg, in 1 study^[12]^ with rosuvastatin 40 mg, and in 1 study^[17]^ with simvastatin 40 mg. Five studies^[12–16]^ included statin-naïve patients, and 3 studies^[17–19]^ included subjects without statin exposure within 14 or 30 days. For hydration, 6 studies^[12,14,16–19]^ used 0.9% sodium chloride, 1 study^[15]^ did 0.9% sodium chloride or half saline, and 1 study^[13]^ did sodium bicarbonate. Four studies^[13–16]^ used oral NAC before and after the procedure, and 3 studies^[17–19]^ did not use oral NAC. Abaci et al^[12]^ reported that about 70% of the subjects used oral NAC. Seven studies^[12,14,16–19]^ used iso-osmolar nonionic contrast media, and 1 study^[15]^ did not specify contrast type. Six studies^[12,15–19]^ defined CI-AKI as an increase in SCr of more than 0.5 mg/dL or more than 25% from the baseline within 72 hours after contrast media exposure, and 1 study^[14]^ was an absolute SCr increase of ≧ 0.5 mg/dL over baseline within 5 days. Quintavalle et al^[13]^ defined CI-AKI as serum cystatin C concentration 10% above the baseline value at 24 hours after administration of contrast media. However, they also reported the rate of an increase in SCr of more than 0.5 mg/dL or more than 25% from the baseline.

**Table 1:**
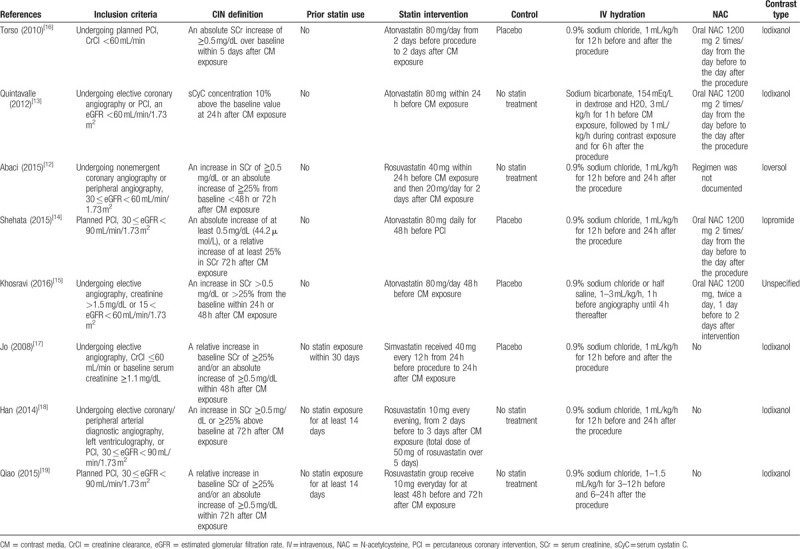
Baseline characteristics and intervention.

Clinical characteristics of included studies are shown in Table 2. There is no significant difference between the statin and control groups in age, sex, DM, or baseline creatinine values. One study^[15]^ did not report the value in age, sex, or DM between the 2 groups, but is described in the text anyway.

**Table 2:**
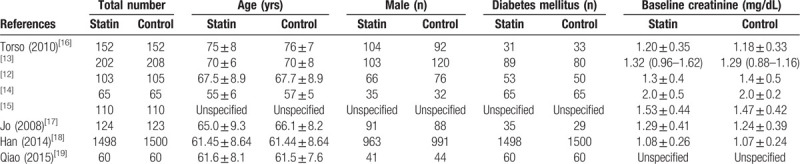
Clinical characteristics.

### 3.3 Assessment of risk of bias

The assessment of the risk of bias for the 8 studies is shown in Supplementary Figure S1. Seven studies were scored as low risk of bias in 5 domains. One study^[15]^ showed some concern about bias arising from the randomization process.

### 3.4 Assessment of statin as a preventive role on CI-AKI

**Figure 2:**
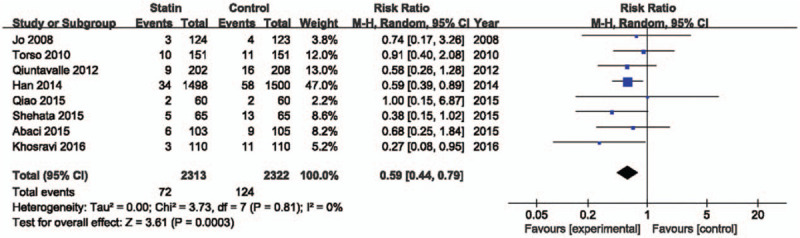
Forest plot of risk ratios with its 95% CI for the incidence of contrast-induced acute kidney injury among patients taking statin vs control. CI = confidential interval.

**Figure 3:**
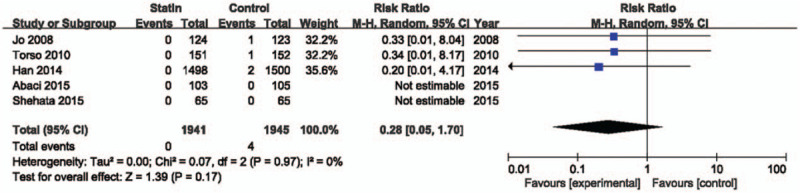
Forrest plot for need for hemodialysis. Forrest plot of pooled data from studies showing the relative risk ratio of the development of contrast-induced acute kidney injury requiring hemodialysis. CI = confidential interval.

We compared the risk of developing CI-AKI in 8 studies that included a total of 2313 subjects in the statin-treated groups and 2322 in the control groups (Fig. 2). There was a significant benefit associated with statin treatment (RR = 0.59; 95% confidential interval [CI] 0.44–0.79; *P* = .0003, *I*^2^ = 0%). Five studies^[12,14,16–18]^ reported an incidence ofacute kidney injury requiring hemodialysis (Fig. 3). The incidence of hemodialysis was low after contrast administration in the statin-treated group, but the reduction was not significant (RR = 0.28; 95% CI 0.05–1.70; *P* = .17, *I*^2^ = 0%).

### 3.5 Subgroup analysis

**Figure 4:**
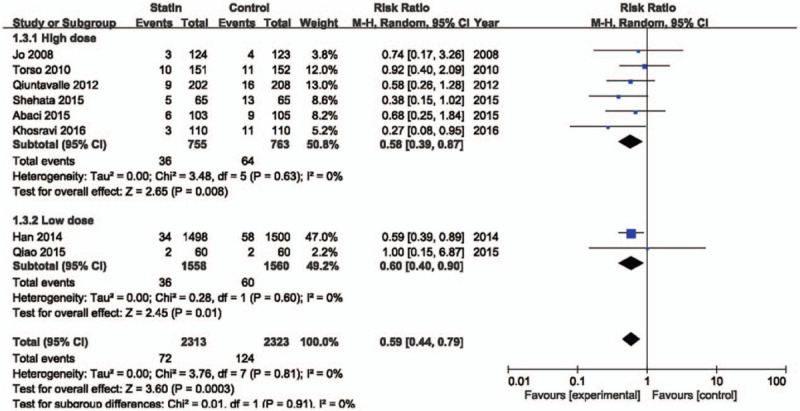
Forest plot of risk ratios with its 95% CI for the incidence of contrast-induced acute kidney injury among patients taking statin vs control base on statin dose. CI = confidential interval.

**Figure 5:**
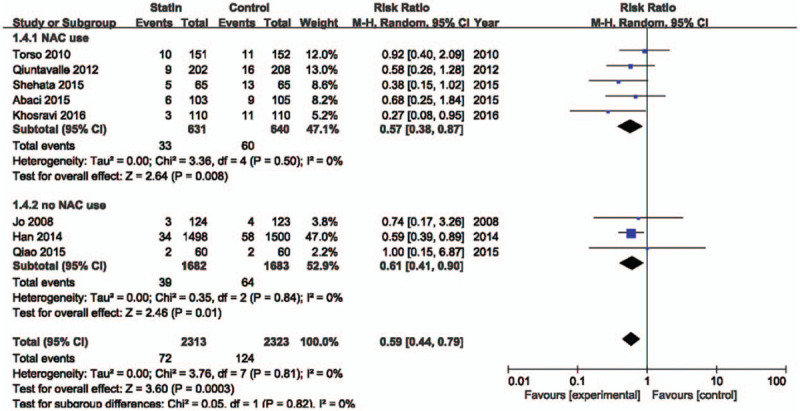
Forest plot of risk ratios with its 95% CI for the incidence of contrast-induced acute kidney injury among patients taking statin vs control base on use of N-acetylcysteine. CI = confidential interval, NAC = N-acetylcysteine.

**Figure 6:**
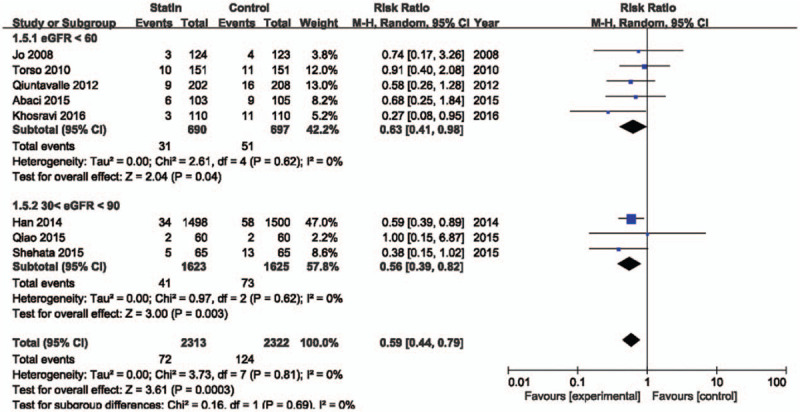
Forest plot of risk ratios with its 95% CI for the incidence of contrast-induced acute kidney injury among patients taking statin vs control base on renal function. CI = confidential interval, eGFR = estimated glomerular filtration rate.

There are interventional and clinical diversities in the included studies. Therefore, we did subgroup analyses based on reported data. Figure 4 shows the meta-analysis based on dose of statin. The high-dose statin-treated group included 6 studies^[12–17]^ treated with atorvastatin 80 mg, rosuvastatin 40 mg, and simvastatin 40 mg. The low-dose statin-treated group included 2 studies^[18,19]^ with rosuvastatin 10 mg. Statin treatment showed a significant beneficial effect on CI-AKI in high (RR = 0.58; 95% CI 0.39–0.87; *P* = .008, *I*^2^ = 0%) and low (RR = 0.60; 95% CI 0.40–0.90; *P* = .01, *I*^2^ = 0%) dose groups. In patients without oral NAC therapy, statin reduced the risk of contrast induced nephropathy by 43% (RR = 0.57; 95% CI 0.38–0.87; *P* = .008) (Fig. 5). In patients who were treated with oral NAC, statin reduced the risk of CI-AKI by 39% (RR = 0.61; 95% CI 0.41–0.90; *P* = .01). The effect of statins on prevention of CI-AKI between both groups was not statistically different (*P* = .82). Definition of renal impairment in the inclusion criteria differed among the studies. Therefore, we analyzed the studies based on criteria of eGFR or creatinine clearance (Fig. 6). Regardless of baseline renal function, statin treatment showed a beneficial effect on prevention of CI-AKI.

### 3.6 Assessment of publication bias and sensitivity analysis

**Figure 7:**
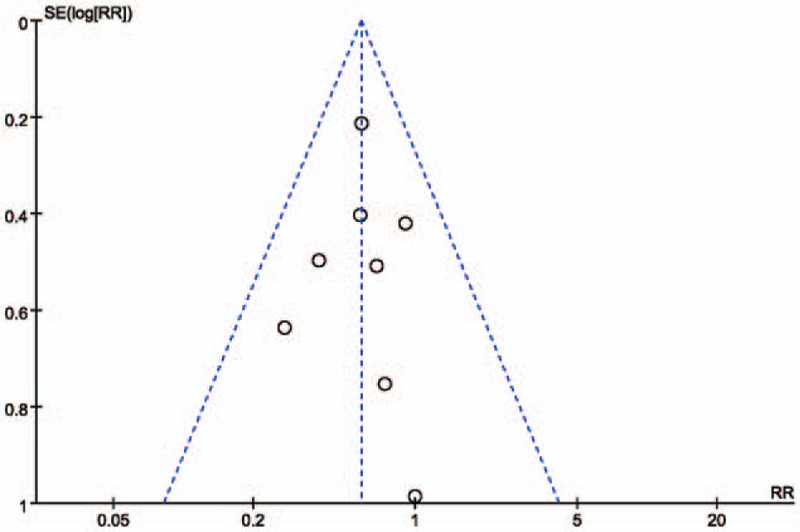
Funnel plot showing publication bias. RR = relative risk.

Publication bias was assessed by Funnel plot (Fig. 7) with a standard error of log RR against RR. It shows the symmetrical distribution of the plot around the summary effect size, showing no publication bias. Sensitivity analysis was done to evaluate the stability of our results. In sensitivity analysis, when individual studies were removed from each of the above-described analyses, the overall RR of CI-AKI demonstrated similar results (Supplementary Figure S2–9).

## 4 Discussion

The present meta-analysis demonstrated that statin pretreatment significantly reduced the incidence of CI-AKI in patients with preexisting renal dysfunction undergoing coronary angiography or PCI, compared with the control (placebo or no statin treatment). The beneficial effect of statin was also observed in the subgroup of patients with CKD stage 3, eGFR <60 mL/min/1.73 m^2^. The effect was consistent regardless of the dose of statin or concomitant use of NAC.

Preexisting renal dysfunction is probably the most important predictor of CI-AKI that occurs up to 25% of patients with CKD.^[20,21]^ CI-AKI in these high-risk patients is associated with prolonged hospitalization, need for dialysis, and increased morbidity and deaths.^[22]^ Therefore, strategies to decrease the occurrence of CI-AKI is urgently needed, especially for these high-risk CKD patients. Previous meta-analysis that aimed to assess the role of statin use in CI-AKI prevention included patients with both CKD and normal kidney function.^[8–11]^ In our analysis, we only included patients with preexisting CKD. Beneficial effect of statin on prevention of CI-AKI is consistent regardless of eGFR category.

Current guidelines recommend adequate hydration before and following contrast exposure, use of iso or low osmolar contrast agents, and reducing the volume of contrast agents to prevent occurrence of CI-AKI.^[23,24]^ However, no consensus about the comparative efficacy of all potential preventive measures against CI-AKI could be drawn. Although current guidelines have not recommended statin pretreatment as a CI-AKI prevention strategy, many researchers have shown that the pleiotropic effect of statins plays a renoprotective role in the prevention of CI-AKI.^[13,25,26]^ The pleiotropic effect includes anti-inflammatory, antithrombotic, and antioxidative effects.^[7,27]^ These pleiotropic effects could attenuate renal cell injury after iodinated contrast exposure. In addition, statins may reduce tubular reabsorption of contrast media, mitigating their direct toxic effects on the renal tubules.^[28]^

In our subgroup analyses, the beneficial effect of pretreatment with statin was consistently observed. Because high-dose statin confers more potent anti-inflammatory effects, previous studies showed that the effect of high-dose statin was stronger than that of a placebo^[14,15]^ or low-dose statin.^[29,30]^ In addition, a recent study suggested that statin plus NAC plus intravenous saline might be the best treatment for CI-AKI prevention.^[31]^ In our analysis, low-dose statin also significantly reduced the incidence of CI-AKI than did a placebo, and a protective role of statin was constantly observed regardless of concomitant use of NAC in CKD patients.

In addition to preexisting renal dysfunction, risk factors for CI-AKI include advanced age, DM, congestive heart failure, use of a large amount of contrast media, and concurrent use of nephrotoxic agents.^[4]^ Recently, several meta-analyses were conducted to assess the efficacy of multiple pharmacological interventions in a general population^[31]^ or high-risk population.^[32,33]^ Our findings are consistent with results from recent meta-analyses, which reported a significant reduction of the occurrence of CI-AKI with statin pretreatment in the high-risk population.^[32,33]^ However, the definition of high-risk patients is heterogeneous between studies, and definition of CKD was not clearly defined in some studies.^[32]^ Compared with these previous reports, we included only subjects with preexisting CKD, who are at high risk of developing CI-AKI. Furthermore, study heterogeneity was low, and subgroup analyses were mostly consistent with the primary outcome, all of which support the robustness of our results.

The present meta-analysis has several limitations. First, we included only 5 studies with eGFR < 60 mL/min/1.73 m^2^, and were not able to analyze patients with eGFR < 30 mL/min/1.73 m^2^. Second, treatment protocol, definitions of CI-AKI, and type of statin were not the same in all studies and differed in postprocedural time period. Third, the pooled analysis of statin vs control showed a significant benefit in the reduction of CI-AKI. However, several individual studies showed nonsignificant outcomes. When they are combined in a pooled summary RR ratio, the result was significant. Caution should be taken when interpreting this result.

In conclusion, the present study demonstrated that statin pretreatment in patients with mild to moderate renal insufficiency reduced the incidence of CI-AKI. The results were consistent regardless of baseline eGFR, dose of statin, or use of oral NAC. During the analysis, we found that most studies of statin pretreatment for prevention of CI-AKI were done with patients undergoing coronary angiography or PCI. Further studies about the effect of statin for prevention of CI-AKI in patients undergoing computed tomography might be needed.

## Author contributions

**Conceptualization:** AJin Cho, Seo Young Sohn.

**Data curation:** Seo Young Sohn.

**Formal analysis:** AJin Cho.

**Investigation:** Seo Young Sohn.

**Methodology:** Seo Young Sohn.

**Supervision:** Young-Ki Lee.

**Validation:** Seo Young Sohn.

**Writing – original draft:** AJin Cho, Seo Young Sohn.

**Writing – review & editing:** AJin Cho.

AJin Cho orcid: 0000-0001-7097-7026.

## Supplementary Material

Supplemental Digital Content

## Supplementary Material

Supplemental Digital Content
