# Supplementary material for: Beneficial effect of statin on preventing contrast-induced acute kidney injury in patients with renal insufficiency: A meta-analysis
Source: Medicine (Baltimore). 2020 Mar 6;99(10):e19473. doi: 10.1097/MD.0000000000019473 (PMC7478506; doi:10.1097/MD.0000000000019473)
Supplement: Supplemental Digital Content [file medi-99-e19473-s001.docx]

S1 Table. Search strategies and detailed records

PUBMED:

#1 Coronary Angiography

#2 Contrast Media

#3 Angioplasty

#4 Acute Coronary Syndrome

#5 Percutaneous Coronary Intervention

#6 #1 OR #2 OR #3 OR #4 OR #5

#7 Hydroxymethylglutaryl-CoA Reductase Inhi

#8 Simvastatin

#9 Atorvastatin Calcium

#10 Rosuvastatin Calcium

#11 #7 OR #8 OR #9 OR #10

#12 #6 AND #11

#13 “Radiocontrast media” [TIAB] OR “radiocontrast agent” [TIAB] OR “Angiography” [TIAB] OR “Angiographies” [TIAB] OR “Coronary Angiographies” [TIAB] OR “Angioplasties” [TIAB] OR “Percutaneous Transluminal Angioplasty” [TIAB] OR “Acute Coronary Syndromes” [TIAB] OR “Percutaneous Coronary Interventions” [TIAB] OR “Percutaneous Coronary Revascularization” [TIAB]

#14 “HMG-CoA Reductase Inhibitors” [TIAB] OR “Statins” [TIAB] OR “statin” [TIAB] OR “Pravastatin” [TIAB] OR “Cerivastatin” [TIAB] OR “Fluindostatin” [TIAB] OR “Fluvastatin” [TIAB] OR “Atorvastatin” [TIAB] OR “Rosuvastatin” [TIAB]

#15 #13 AND #14

#16 #12 OR #15

#17 (groups[TIAB] OR trial[TIAB] OR randomly[TIAB] OR "drug therapy"[SH] OR placebo[TIAB] OR randomized[TIAB] OR "controlled clinical trial"[PT] OR "randomized controlled trial"[PT]) NOT (animal[MH] NOT (humans[MH] AND animals[MH]))

#18 #16 AND #17

EMBASE:

#1 ‘Radiocontrast media’:ab,ti OR ‘radiocontrast agent’:ab,ti OR ‘Angiography’:ab,ti OR ‘Angiographies’:ab,ti OR ‘Coronary Angiographies’:ab,ti OR ‘Angioplasties’:ab,ti OR ‘Percutaneous Transluminal Angioplasty’:ab,ti OR ‘Acute Coronary Syndromes’:ab,ti OR ‘Percutaneous Coronary Interventions’:ab,ti OR ‘Percutaneous Coronary Revascularization’:ab,ti

#2 ‘HMG-CoA Reductase Inhibitors’:ab,ti OR ‘statins’:ab,ti OR ‘statin’:ab,ti OR ‘Pravastatin’:ab,ti OR ‘cerivastatin’:ab,ti OR ‘fluindostatin’:ab,ti OR ‘Fluvastatin’:ab,ti OR ‘Atorvastatin’:ab,ti OR ‘Rosuvastatin’:ab,ti

#3 'crossover procedure'/exp OR 'double blind procedure'/exp OR 'randomized controlled trial'/exp OR 'double blind procedure'/exp OR random* OR factorial* OR crossover* OR 'cross over' OR 'cross-over' OR placebo* OR (doubl* AND blind*) OR (single* AND bind*) OR assign* OR allocat* OR volunteer*

#4 #1 AND #2 AND #3

WEB OF SCIENCE:

#1 “Radiocontrast media” OR “radiocontrast agent” OR “Angiography” OR “Angiographies” OR “Coronary Angiographies” OR “Angioplasties” OR “Percutaneous Transluminal Angioplasty” OR “Acute Coronary Syndromes” OR “Percutaneous Coronary Interventions” OR “Percutaneous Coronary Revascularization” OR “Contrast Media” OR “Coronary Angiography” OR “Angioplasty” OR “Acute coronary syndrome” OR “Percutaneous coronary intervention”

#2 “HMG-CoA Reductase Inhibitors” OR “statins” OR “statin” OR “Pravastatin” OR “cerivastatin” OR “fluindostatin” OR “Fluvastatin” OR “Atorvastatin” OR “Rosuvastatin OR “Hydroxymethylglutaryl-CoA Reductase Inhibitors” OR “Simvastatin” OR “Atorvastatin calcium “OR “Rosuvastatin calcium”

#3 TOPIC:("randomized controlled trial" OR "controlled clinical trial" OR randomized OR randomly)

#4 #1 AND #2 AND #3
