# Supplementary figures and images for: Beneficial effect of statin on preventing contrast-induced acute kidney injury in patients with renal insufficiency: A meta-analysis
Source: Medicine (Baltimore). 2020 Mar 6;99(10):e19473. doi: 10.1097/MD.0000000000019473 (PMC7478506; doi:10.1097/MD.0000000000019473)

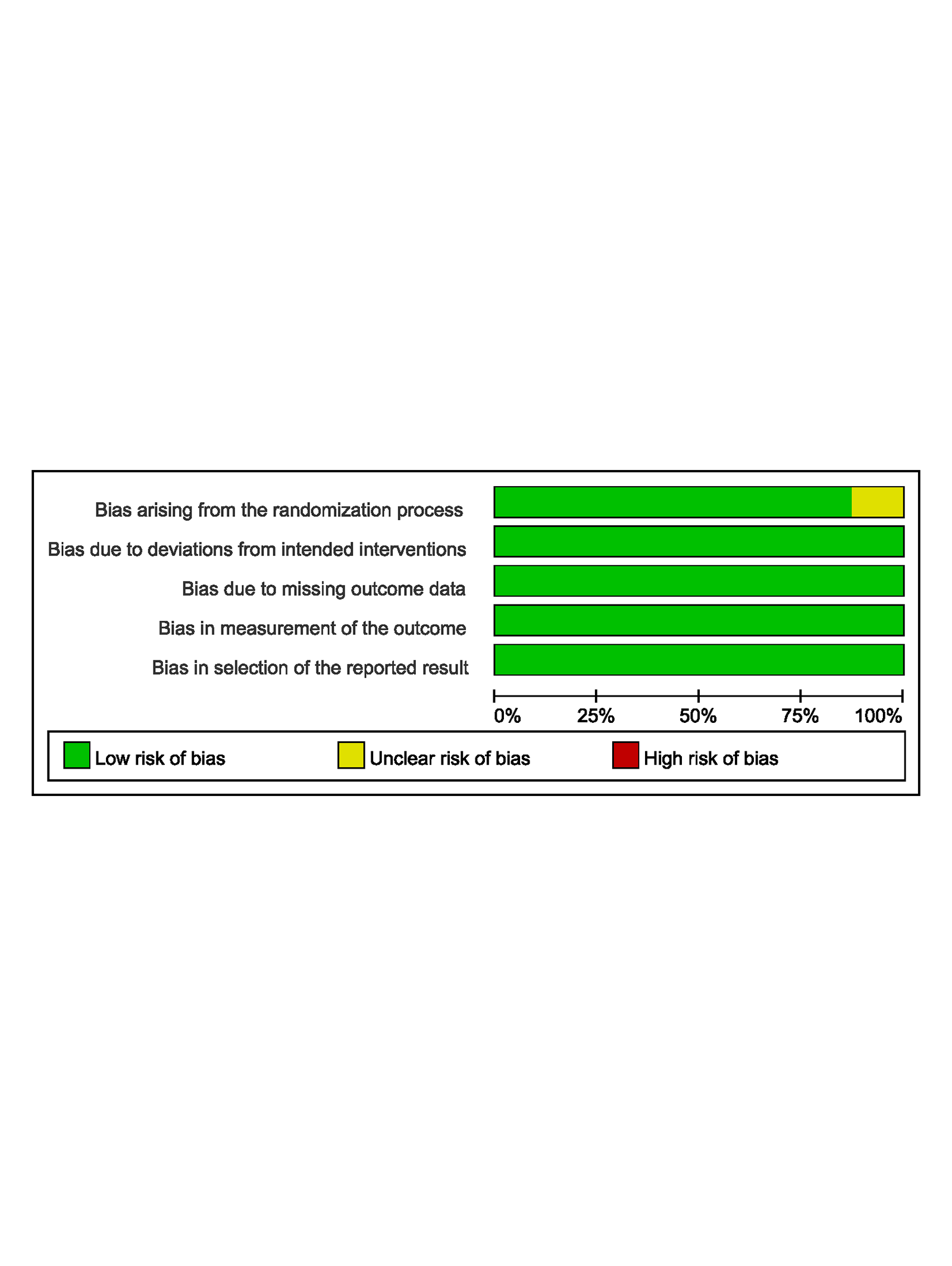

Supplement: Supplemental Digital Content [file medi-99-e19473-s002.jpg]

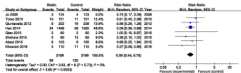

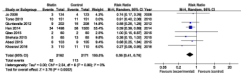

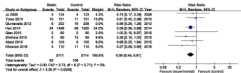

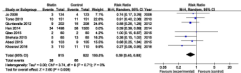

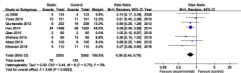

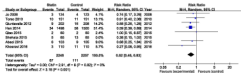

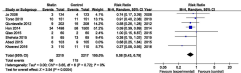

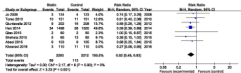

Supplement: Supplemental Digital Content [file medi-99-e19473-s003.pdf]
